# Some Ancient Theories

**Published:** 1891-04

**Authors:** H. H. Schumann

**Affiliations:** Chicago


### SOME ANCIENT THEORIES.

BY H. H. SCHUMANN, D.D.S., CHICAGO.

So much has appeared of late in the various dental journals in regard to the etiology of dental irregularities, that a few remarks in regard to some "theories" may not be out of place. I cannot understand why certain theories are constantly brought up again and again in dental societies, and valuable time lost in the discussion of things which ought not to need any further explanations. I want in these few lines simply to enumerate a few of the former theories in regard to this subject, and some of the reasons why I think them at present useless, doing this simply to enlighten a few who seem not to have kept up with the times, so that the valuable few moments allowed in our meetings be not wasted in settling wild conclusions, and also to form a thread for discussion of a few unsettled questions.

Most authors satisfy themselves in their works on oral deformities by quoting a great number of appliances, both complicated and simple, and do not enter into theory very deeply, in that respect being far behind our professional brothers and writers abroad. I think if they would venture to apply their knowledge on this subject they would be far more valuable to the student and the profession in general. If a practitioner knows the cause, signs, and symptoms of mechanical conditions, it would, as a rule, be very easy for him to suggest means by which the disturbance could be remedied much easier, quicker, and surer than if he has to remember a lot of empirical measures, none of which usually will fit his particular case.

Dr. Eugene Talbot has written a book of only one hundred and fifty pages, but to my mind it conveys more practical knowledge than any book on dental deformities published up to date, no matter how large or how complete it may be; and I am of the opinion that his book gives all the practical knowledge necessary, simply because those few pages are saturated with good, sound, sensible ideas, and not with modes of treatment.

In enumerating the following theories, I will state, in the beginning, that, to my mind, those elucidated in the book mentioned are the clearest and most sensible ones, and based on more facts than any I know of.

Dr. Cartwright, many years past, wrote the first able article on etiology in England. It is true that most of the knowledge we have on this subject was given to the profession by English writers. Dr. Cartwright says correctly, in his paper, that irregularities of the teeth are not found in any of the aboriginal tribes, as the Japanese, Chinese, or African negroes; but he cannot account for the fact that it is found in the nobility of England. American authors have since explained this condition as being due to the non-observance of selective marriage; through intermarriage of blood relations the physiological powers of both sides become greatly diminished by their high grade of civilization, overtaxed brains, and nervous systems. It may also be due to the intermarriage of persons afflicted with transmissible diseases resulting from debauchery.

Another valuable writer on the subject of dental deformities was Dr. Langdon Down, of England, although his theories to-day have been replaced by better ones. He says in his works that especially the V- and saddle-shaped arches are more frequently found with the idiotic than in people in a sane condition. Talbot and others, within the past few years, have, in regard to these conditions, made personal observations of hundreds of idiots and feeble-minded subjects throughout this country, but found that such conditions do not *per se* indicate idiocy, and that among them only fifty per cent. could be classed as having contracted dental arches, while fully fifty per cent. displayed large and well-formed jaws. Talbot found a greater percentage of V-shaped arches among the deaf, dumb, and blind than among the idiotic.

An old theory is that of Dr. Ballard, one which is constantly receiving attention even in these days. He holds the belief that the V-shaped arch is due to thumb-sucking. He also says it is found mostly in idiots; yes, he even goes so far as to say that children, sucking their thumbs, show therein a predisposition to idiocy. It is well known that a child, if it suck its thumbs, will not hold it directly in the centre of the mouth, and will therefore not produce the V-shaped type of irregularity, but generally holds the left thumb somewhat to that side, and the right to its corresponding side. Thus, if thumb-sucking would produce any deformity, it would not push out the central incisors, and surely not towards a cutting edge, as they are usually found in V-shaped arches, and somewhat twisted. Further, if this theory of Dr. Ballard be correct, why does thumb-sucking not affect the temporary teeth? Those teeth come during the period of thumb-sucking, and children generally stop this habit before they reach the age of eight or ten years. The fact is that the temporary teeth are hardly ever found irregular, and I have never heard of a V-shaped arch in a child's mouth.

Another able writer is Dr. Coles, but his theories are also old, and have been discussed so often that a constant rediscussing is a waste of valuable time in dental meetings for those who read and keep up their studies. Dr. Coles thinks that high arches are mostly found in small-skulled idiots,—that is, in creatures whose skulls are ossified in a very early period of life, due to an inflammation and hypertrophied condition of the brain membrane *in utero* or soon after birth. This is confined mostly to the bones forming the base of the skull, and more particularly to the sphenoid bone. Dr. Coles says that the early ossification and contraction of the bones tend also to draw in the maxillary bones and their alveolar border. If we now examine a skull, we will find that the floor of the nares is on a level with the condyles of the occipital bone, but the roof of the nasal cavity is on a level with the sphenoid. The palate, then, is entirely out of the range of the sphenoid. Now, if this bone (sphenoid) does not contract, it may cause some contraction of the nose, but not of its floor or the roof of the mouth. I cannot see how a contraction of the upper part of the nares could possibly cause an anterior protrusion of the superior maxillary alveolus. But I can very well understand that a long superior alveolar border produces at the same time a high vault, and the latter is Dr. Talbot's explanation of a high palate.

Dr. Tomes says, in his work on oral surgery, that irregularity, especially the saddle-shaped arch, is due to tonsillitis or some other throat-trouble, causing the child to breathe through the open mouth. His explanation of the results is as follows: By opening the mouth the buccinator muscles are drawn tense, thus pressing on the developing teeth. Now, the buccinator muscle lies in the cheek, and presses uniformly on the whole posterior part of the alveolar border of both upper and lower jaws. How, then, can it, when drawn tightly on the whole line of teeth (both jaws), cause a dropping in of three or four, or sometimes a single upper tooth, and at times even on one side of the jaw only. This theory is old, very old, and has been discussed and thoroughly defeated time and time again, and still it has adherents, and is brought up again and again. Only yesterday I read in the INTERNATIONAL DENTAL JOURNAL a paper on adenoid hypertrophy, an essay read and discussed before such an able body of men as the New York Odontological Society. The effects of adenoid growths, the theory advanced both in the paper and in the discussion, is nothing more nor less than the theory of Dr. Tomes,—mouth-breathing. How such a body of men could take all those evolutions for granted, discuss and fully believe in the plausibility of that theory at this date, is more than I can understand. How many persons suffering from V-shaped arches have adenoid growths? How do the believers in the mouth-breathing theory account for arches that are V-shaped on one side only? Have any of them ever seen a single jaw developed symmetrically alike on both sides? How is it, then, that they can still believe in such theories?

Another theory of dental irregularity is that of Dr. Kingsley, who ascribes irregularities to hereditary diathesis. If that were true, the child would have exactly the same irregular development as its parents. In practice, though, that is not often found to be the case.

When I hear of such old theories still discussed, still believed in, it appears to me as if a great many professional brethren do not read new works. I think that this is especially true of the young men in the profession. Dental meetings are always short, and time is too valuable to be spent for the benefit of the lazy and thoughtless class of men. Of all the subjects most neglected by that class in their readings and study, dental etiology seems to be the one. It is true, it requires a little more careful thought and more work to keep abreast with the time. A little reading and thinking will do a great deal in this line, especially to render discussions more fluent and more valuable, and it is about time that such old and far-fetched theories as some of those spoken of should be dropped, and the newer, simpler, and more reasonable ones carried out more fully.

